# Simultaneous Monitoring of Sweat and Interstitial Fluid Using a Single Wearable Biosensor Platform

**DOI:** 10.1002/advs.201800880

**Published:** 2018-08-02

**Authors:** Jayoung Kim, Juliane R. Sempionatto, Somayeh Imani, Martin C. Hartel, Abbas Barfidokht, Guangda Tang, Alan S. Campbell, Patrick P. Mercier, Joseph Wang

**Affiliations:** ^1^ Department of Nanoengineering University of California San Diego, La Jolla CA 92093 USA; ^2^ Department of Electrical and Computer Engineering University of California San Diego, La Jolla CA 92093 USA

**Keywords:** interstitial fluid, iontophoresis, sweat, tattoo biosensors, wearable electronics

## Abstract

The development of wearable biosensors for continuous noninvasive monitoring of target biomarkers is limited to assays of a single sampled biofluid. An example of simultaneous noninvasive sampling and analysis of two different biofluids using a single wearable epidermal platform is demonstrated here. The concept is successfully realized through sweat stimulation (via transdermal pilocarpine delivery) at an anode, alongside extraction of interstitial fluid (ISF) at a cathode. The system thus allows on‐demand, controlled sampling of the two epidermal biofluids at the same time, at two physically separate locations (on the same flexible platform) containing different electrochemical biosensors for monitoring the corresponding biomarkers. Such a dual biofluid sampling and analysis concept is implemented using a cost‐effective screen‐printing technique with body‐compliant temporary tattoo materials and conformal wireless readout circuits to enable real‐time measurement of biomarkers in the sampled epidermal biofluids. The performance of the developed wearable device is demonstrated by measuring sweat‐alcohol and ISF‐glucose in human subjects consuming food and alcoholic drinks. The different compositions of sweat and ISF with good correlations of their chemical constituents to their blood levels make the developed platform extremely attractive for enhancing the power and scope of next‐generation noninvasive epidermal biosensing systems.

## 1 Introduction

Wearable devices offer the exciting ability to monitor the physiologic state of humans in a real‐time manner. To be practical, wearable sensors must be comfortable, unobtrusive, and compliant with the soft features of the human body. Significant recent efforts have thus been made toward the development of soft, epidermally mounted systems.^1^, ^2^, ^3^ At the same time, wearable devices should monitor interesting and/or actionable parameters, and thus, significant research activity has augmented the measurement of vital signs and motion with the monitoring of biochemical markers.^4^, ^5^, ^6^, ^7^, ^8^ Recent efforts have focused on epidermal biomonitoring systems of two readily obtainable biofluids: skin interstitial fluid (ISF)^9^, ^10^, ^11^, ^12^ and sweat.^13^, ^14^, ^15^, ^16^, ^17^, ^18^, ^19^ Reliable and convenient noninvasive monitoring of target biomarkers in these biofluids can potentially have a major impact on a wide variety of healthcare and wellness applications. For example, the detection of glucose in sweat and ISF has received a tremendous recent attention in connection to the management of diabetes.^9^, ^20^, ^21^, ^22^, ^23^

Sweat is an epidermally available biofluid containing metabolites, electrolytes, trace elements, and low levels of macromolecules (e.g., proteins).^24^ Several of these biomarkers have been shown to reflect the physiological status of blood, as they diffuse from the bloodstream through sweat and hence reflect the wearer's health and fitness. The ease of access to numerous sampling sites makes sweat a particularly attractive medium for noninvasive biomarker monitoring. However, sweat can only be analyzed once it is excreted to the outer skin surface. As a result, several sweat generation processes have been introduced, including prolonged exercise, thermal heating, stress, and iontophoretic stimulation.^25^ Iontophoresis (IP) is an established process used to induce the ion/molecular flow by applying a mild electric current across the skin and is widely used in clinics for diagnostic and therapeutic purposes. Alternatively, the ISF resides under the skin surface and fills the interstitial space between tissue cells, but also contains clinically relevant biomarkers. Many ISF constituents display good correlation to concurrent blood levels, as they diffuse through the endothelium directly from blood vessels.^9^, ^10^, ^23^, ^26^ Similar to biomarker detection in sweat, ISF analysis requires access to the biofluid itself. Noninvasive ISF sampling can be readily performed on the epidermis by IP and ultrasound (sonophoresis).^27^, ^28^ Despite the beneficial features of each biofluid, noninvasive epidermal biomarker monitoring has been limited to separate analyses of sweat or ISF using different wearable devices.

Due to the attractiveness of ISF and sweat as noninvasive biofluids, simultaneous analysis of these epidermally sampled biofluids is highly desired. Such parallel sweat and ISF monitoring could expand the scope of detecting biomarkers and improve clinical accuracy. However, to date, dual sampling/analysis of ISF and sweat in real‐time has not been demonstrated, largely due to the different approaches used to sample these biofluids. Since both biofluids can be obtained on the epidermis, sweat and ISF can share the same sampling sites at the skin surface. However, most common methods of sweat stimulation (e.g., exercise and thermal heating) involve uncontrolled sweat generation locations. Use of these sweat generation methods, coupled with ISF sampling procedures for their simultaneous analyses, would lead to extensive intersample mixing of the two biofluids, and could thus cause large interferences in the biosensing signals. This challenge can be addressed through utilization of IP and reverse IP that enable controlled, on‐demand localized sampling of sweat and ISF at rest, respectively, onto or across the skin.^13^, ^20^, ^29^, ^30^, ^31^ The use of IP for electrically inducing migration of ions/molecules across the skin surface can be used to both deliver and extract desired compounds. Iontophoretic transdermal drug delivery is used to distribute agonist drugs (e.g., pilocarpine or lidocaine) from an electrode across the skin.^30^, ^31^, ^32^ For instance, when administered into skin, pilocarpine induces localized sweat generation. In contrast, the use of IP to draw molecules (e.g., glucose, urea, alcohol, etc.) through the epidermis to the skin surface, known as reverse iontophoresis, enables noninvasive sampling of ISF constituents.^9^, ^20^, ^23^, ^33^, ^34^

**Figure 1:**
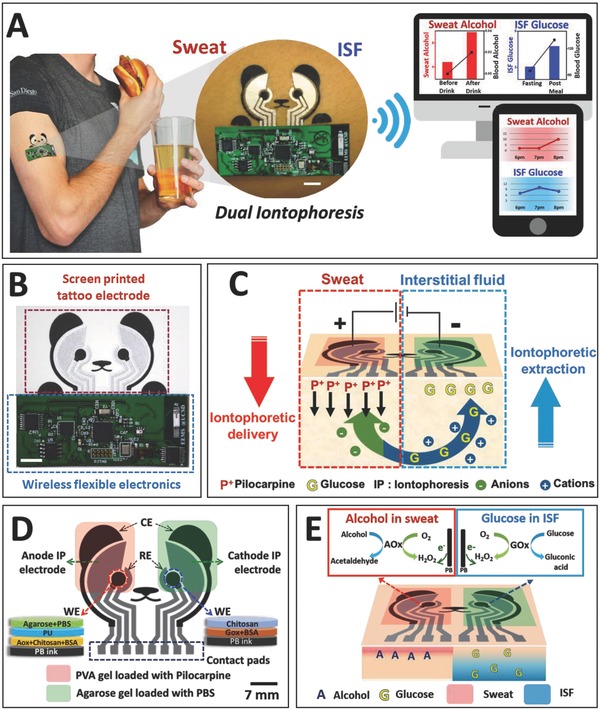
The concept of simultaneous noninvasive sampling and monitoring of ISF and sweat. A) Depiction of wearable iontophoretic biosensor device on a printed tattoo platform for glucohol (glucose + alcohol) sensing on a human subject, along with wireless real‐time transmission of the ISF glucose and sweat alcohol response. Scale bar: 7 mm. B) Image of the screen‐printed glucohol biosensor coupled with wireless flexible printed circuit board. Scale bar: 7 mm. C) Schematic representation of iontophoretic operation. Induced generation of alcohol‐containing sweat by iontophoretic delivery of pilocarpine at the anode with simultaneous reverse iontophoretic sampling of ISF glucose at the cathode. D) Schematic representation of glucohol biosensor, showing the “panda‐like” electrode layout with iontophoretic gels and layered composition of the working electrodes. Scale bar: 7 mm. E) Schematic representation of biosensing operation. Amperometric detection of alcohol in the generated sweat and of glucose in the extracted ISF using AOx and GOx, respectively, along with measurement of the hydrogen peroxide product of these enzymatic reactions.

Herein, we report on the development of a new class of wearable biomonitoring device that enables, for the first time, simultaneous and yet independent sampling and analysis of two epidermal biofluids (ISF and sweat) in a single device (**Figure** **1**A). This dual sampling and detection epidermal system has been realized through the parallel operation of reverse iontophoretic ISF extraction across the skin and iontophoretic delivery of a sweat‐inducing drug (pilocarpine) into the skin at separate locations. The printed, tattoo‐like flexible iontophoretic system has been integrated with electrochemical biosensors to enable simultaneous real‐time analysis of the different sampled biofluids. These biosensors were designed to electrochemically measure ISF glucose at the cathode side (using a glucose oxidase (GOx)‐based biosensor) and sweat alcohol at the anode side (using an alcohol oxidase (AOx)‐based biosensor) as model analytes, yielding a glucohol (glucose + alcohol) wearable epidermal platform. Such wearable system has been fabricated on epidermal temporary tattoo platform through cost‐effective screen‐printing technique for disposable single use. The developed simultaneous detection of glucose and alcohol offers considerable promise for a variety of practical applications. A flexible circuit board was magnetically attached to the glucohol tattoo to drive the iontophoretic electrodes, control the biosensors, and wirelessly transmit the sensed information to a smartphone for practical self‐monitoring applications (Figure 1B). The integrated wireless electronics were programmed to perform the sequential operations of IP and amperometric measurements with no‐apparent cross‐talk between these functional sampling and detection steps. Furthermore, the attractive performance and practicality of the new epidermal tattoo iontophoretic sensor was demonstrated with human subjects consuming glucose and alcohol. Such successful simultaneous on‐demand sampling (at rest) and analysis of ISF and sweat on a single wearable device hold considerable promise for enhancing the capabilities and scope of noninvasive epidermal biomarker detection platforms.

## 2 Results and Discussion

### 2.1 Principle of Dual Iontophoresis for Simultaneous Sampling of ISF and Sweat

We report on the development and characterization of a dual iontophoretic biosensor applied for the noninvasive monitoring of glucose and alcohol. Our objective was to combine sweat and ISF collection processes using a single wearable device for obtaining dual‐fluid biomonitoring applications. An attractive feature of this new biosensing platform is the simultaneous, on‐demand, and localized generation and collection of two noninvasive biofluids (ISF and sweat) at rest along with integration of a dual electrochemical biosensing system for detecting ISF glucose and sweat alcohol on a single disposable wearable tattoo platform. The dual iontophoretic system combining the two iontophoretic operations was demonstrated, for the first time, with iontophoretic ISF extraction at the cathode compartment and iontophoretic delivery of the sweat‐inducing pilocarpine agent at the anode compartment (Figure 1C). Both IP procedures occurred simultaneously based on two main mechanisms: (1) electrorepulsion: ions with the same charge as the iontophoretic electrode are repelled and oppositely charged ions are attracted. On the electrode surface, the anode repels the positively charged cations (e.g., pilocarpine), while attracting negatively charged anions (e.g., Cl^−^, ascorbate, urate) from the dermis and epidermis. On the cathode side, anions are repelled while cations (e.g., Na^+^) are attracted to the surface. (2) Electro‐osmosis: convective flow from the anode to cathode, including neutral molecules (e.g., glucose and urea) induced by the flow of charged particles. The type of IP, either delivery or extraction, is determined by the direction of the induced major flow of the molecule of interest through the skin.

In our wearable system, the tattoo iontophoretic patch delivers the positively charged pilocarpine drug at the anode by electrorepulsion (iontophoretic delivery), allowing the delivered drug to generate sweat, which is localized at the pilocarpine loaded gel in the anode compartment, as shown in Figure 1C. Simultaneously, the major convective flux occurs from the anode to cathode under the skin, as dominated by the flow of counterions (mainly Na^+^). Cationic electro‐osmotic flux toward the cathode is due to the overall negative charge of human skin, leading to the migration of positive ions and of neutral molecules, such as glucose (iontophoretic extraction, reverse IP). Such reverse iontophoretic extraction of ISF glucose obviates interference issues by charge and size exclusion during the extraction process as only small ions or molecules can be extracted effectively, excluding proteins and many other biomolecules. In addition, major electroactive species in biofluids, such as ascorbate and urate, can be extracted only at the anode due to their negative charge. Accordingly, the new dual‐biofluid iontophoretic system allows selective, simultaneous, and localized on‐demand sampling at each iontophoretic electrode, with sweat fluid on the anode, and ISF fluid on the cathode, with no intersample mixing. The target sweat and ISF biomarkers can thus be detected by placing the corresponding electrochemical biosensors at the anode and cathode sites, respectively.

### 2.2 Glucose and Alcohol as Model Analytes in ISF and Sweat

Glucose and alcohol are two of the most common target analytes for wearable sensing applications owing to their tremendous healthcare and safety importance. The simultaneous detection of alcohol and glucose is particularly important for diabetics, who are more susceptible to hypoglycemia when consuming alcohol. Alcohol consumption contributes to ≈10% of severe hypoglycemia cases in diabetics, particularly those suffering from type 1 diabetes.^35^ Insulin‐dependent diabetics are strongly recommended to consume any alcoholic beverages along with dietary carbohydrates to counteract decreases in blood glucose (BG) induced by alcohol intake, due to this increased risk of hypoglycemia. Even for healthy individuals, excessive alcohol consumption can indirectly trigger obesity, which is a major risk factor for developing noninsulin dependent diabetes (i.e., type 2 diabetes), among other health issues. Excessive alcohol consumption may also lead directly to insulin malfunctions, further increasing the risk of developing diabetes. Accordingly, the tremendous demands for monitoring glucose and alcohol have led to significant efforts toward self‐testing of blood glucose and alcohol levels. During periods of alcohol intake, the concurrent concentrations of alcohol in blood and sweat have been shown to display a close correlation, without extensive time lag or errors commonly exhibited by breath analyzers or transdermal sweat monitoring devices.^36^, ^37^ Similarly, concentrations of glucose in ISF have shown a great correlation to blood levels as glucose diffuses directly from the blood stream into the ISF in order to supply skin tissue cells with necessary nutrients.^9^, ^27^, ^34^ Ultimately, these correlations and issues led to our selection of glucose and alcohol as model analytes for demonstrating the capabilities and potential of the new dual‐iontophoretic sweat/ISF sampling and detection system.

### 2.3 Design/Integration of Dual Iontophoretic Electrochemical Sensor on a Tattoo Platform

The designed iontophoretic device was incorporated with an electrochemical sensing system on a wearable tattoo platform in a panda bear shape. The geometry of each electrode transducer was the most important factor for successful sampling (stimulation/extraction) and sensing on a single shared platform. The electrode layout consisted of anodic and cathodic compartments. Each compartment included an IP electrode (either anode or cathode) and three sensing electrodes (working (WE), reference (RE), and counter (CE)) covered with a single hydrogel that doubled as a biofluid sampling platform and as a storage reservoir for electrochemical sensing (Figure 1D). The IP electrode was positioned in between the sensing WE and CE for effective diffusion of generated biofluids (ISF, sweat) during the sensing operation. All compartment electrodes (IP, WE, RE, CE) were covered with the hydrogel to avoid skin burning during IP operation and eliminate the need for separate procedures for fluid stimulation, sampling, and measurement, which has been a main challenge for conventional iontophoretic devices. Further, the hydrogel was formed using a buffered solution to protect the epidermis from potential pH changes during repeated sensing due to ionic build‐up at the sampling site. However, different hydrogels were utilized at each compartment (anodic/cathodic) for efficient sample collection as each of the IP electrodes had different goals to achieve. The anode delivered the pilocarpine drug, therefore a more porous structured hydrogel was preferred for efficient release of the drug from gel to skin. A cryogel was selected to be the anode drug sorbent based on our previous study,^15^ and was loaded with 1% pilocarpine nitrate solution. The cathode hydrogel required a less porous structure to store the extracted ISF, and hence 4% agarose gel with PBS was utilized at the cathode IP compartment. As a result of IP, the simultaneous generation of sweat and ISF was localized on the corresponding anode and cathode compartments, respectively, and the electrochemical biosensors were integrated to measure alcohol and glucose in the corresponding generated biofluids.

The amperometric alcohol biosensor system at the anode measured alcohol in generated sweat reflecting blood alcohol levels, while the glucose biosensor system at the cathode detected the extracted ISF glucose, correlated to BG levels. Both sensing working electrodes were composed of a screen‐printed Prussian blue (PB) transducer, the enzyme bioreceptor (GOx for glucose, or AOx for alcohol), and chitosan for the enzyme immobilization. Due to the pilocarpine interference on the PB electron shuttling activity, 2% agarose (containing PBS) was applied on top of the PU layer, as demonstrated in our previous work.^15^ The amperometric response was recorded at −0.2 V (vs Ag/AgCl) by monitoring the reduction of the hydrogen peroxide product of the enzymatic reactions at the catalytic PB transducer (Figure 1E), based on the following biocatalytic reactions

(1) $$\mathrm{Glucose}+\mathrm{oxygen}\;\overset{\mathrm{GOx}}{\to }\;\mathrm{hydrogen}\;\mathrm{peroxide}+\mathrm{gluconic}\;\mathrm{acid}$$

(2) $$\mathrm{Alcohol}+\mathrm{oxygen}\;\overset{\mathrm{AOx}}{\to }\;\mathrm{hydrogen}\;\mathrm{peroxide}+\mathrm{acetaldehyde}$$

### 2.4 Mechanical Deformation Studies of Glucohol Sensor

**Figure 2:**
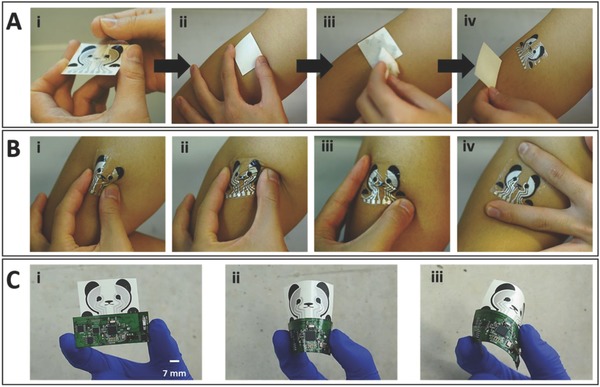
Skin conformability and mechanical integrity of glucohol biosensor. A) Tattoo application process beginning with (i) removal of the primary (nonstick) layer of a double‐sided adhesive. (ii) Placement and (iii) wetting of the tattoo on the upper arm of the subject. (iv) Gradual removal of the damp tattoo paper substrate layer to complete the transfer process. B) Mechanical deformation tests of transferred tattoo, including (i) 180° inward bend, (ii) lateral twisting, (iii) corner stretching, and (iv) vertical stretching. C) (i) Tattoo integrated with wireless flexible PCB followed by additional strain tests, (ii) 180° inward bend, and (iii) rotational bend. Scale bar: 7 mm.

The glucohol tattoo was readily transferred onto the upper arm with ease of use as shown in **Figure** **2**A, and various deformation tests were subsequently carried out in order to investigate the mechanical stability of the device under mechanical strains expected during on‐body operation. Upon application to the arm, the wearable device underwent bending, twisting, and stretching to examine its resistance to such strains and for the presence of possible cracks or breaks in the electrode surface (Figure 2B). This examination indicated no apparent cracks following these deformations and that the tattoo remained in good contact with the skin throughout the test. For practical applications, the tattoo sensor was combined with flexible electronics board and similar (twisting and bending) strains were applied (Figure 2C). These strains caused no apparent structure damage reflecting the conformal character and flexible properties of the entire integrated tattoo device.

### 2.5 Optimization of the Iontophoresis Operational Parameters

**Figure 3:**
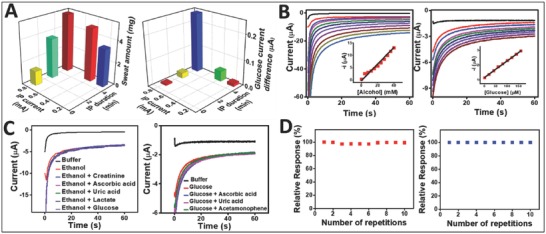
Optimization of iontophoresis and electrochemical sensing of glucohol biosensor. A) Influence of the IP conditions upon the glucose sampling rate (Right) and on the sweat rate (Left). B) Amperometric response of the glucohol biosensor to increasing ethanol concentrations from 0 to 40 × 10^−3^ m in phosphate buffer solution, pH 7.4, with 4 × 10^−3^ m increments (left). Inset: corresponding calibration plot. Amperometric response of the glucohol biosensor to increasing glucose concentrations from 0 to 160 × 10^−6^ m in phosphate buffer solution, pH 7.4, with 20 × 10^−6^ m increments (right). Inset: corresponding calibration plot. C) Amperometric response of glucohol biosensor to common electroactive interferences using ethanol (left) and glucose (right). D) Stability of the response of the glucohol biosensor to repetitive measurements of ethanol (left) and glucose (right). All amperometric measurements were carried out for the duration of 60 s and at a potential step of −0.2 V.

Since high IP current may induce skin irritation,^9^, ^38^ the new wearable iontophoretic system was optimized to minimize the current density and IP period. Further, the use of buffered gel coatings prevented burns from adverse pH effects. The IP efficiency was evaluated by measuring the amount of sweat generated at the anode and the differences in the glucose sensor signal at the cathode (**Figure** **3**A). On the anode, the amount of sweat produced was varied by combining different IP current intensities and durations, clearly showing a tendency that longer periods and higher current values produced larger amounts of sweat. A similar trend was observed for the response to extracted glucose at the cathode. The glucose flux (extracted glucose amount) at the cathode compartment was indirectly measured by comparing the amperometric signals before and after IP. Such signal difference is expected to be proportional to the amount of extracted ISF glucose on the cathode electrode. Applying IP for 5 min at 0.3 mA cm^−2^ showed distinctively higher glucose signals compared to other IP operating conditions (involving lower current levels and shorter periods). The latter resulted in low levels of extracted glucose that approach the detection limit of the glucose biosensor. All subsequent work was thus carried out using a 5 min IP at 0.3 mA cm^−2^, hence ensuring effective generation of sweat and ISF for the sensing of alcohol and glucose.

### 2.6 Characterization of Glucohol Tattoo Biosensor In Vitro

The performance of the tattoo‐based glucose and alcohol biosensors was evaluated first in a buffer medium using their corresponding physiological concentration ranges. Glucose in ISF has similar levels as BG, but only 1/100 of that can reach the skin during the extraction process.^39^ Therefore, the glucose biosensor was evaluated within a 0–160 × 10^−6^ m concentration range with 20 × 10^−6^ m increments, and the observed signal resulted in a highly linear and selective response to glucose concentrations in the presence of relevant electroactive interference (Figure 3B,C). Similarly, the alcohol sensor response was tested over a 0–40 × 10^−3^ m alcohol concentration range and showed well‐defined amperometric signals with 4 × 10^−3^ m alcohol additions even in the presence of electroactive interference (Figure 3B,C). Furthermore, agarose gels on the cathode composed of 0.1 m phosphate buffered solution (PBS) offered resistance to changes of pH and electrolytes in the generated biofluids to minimize impact on enzyme activity. This medium thus offers a robust sensor response, independent of variations in the ISF composition (except the glucose target). On the anode side, sweat induced by pilocarpine has shown less pH variations when compared to sweat obtained through exercise and thermal generation.^40^ The working electrode for the alcohol biosensing was covered with PBS‐containing agarose in order to address the pilocarpine effect on the PB transducer and to offer a consistent electrolyte composition on the working electrode, as described in our earlier study.^15^

### 2.7 Characterization of Integrated Glucohol Tattoo Biosensor with Human Subjects

**Figure 4:**
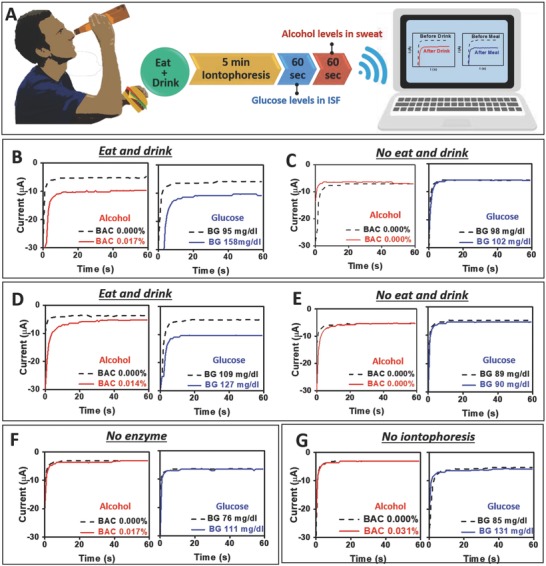
Electrochemical characterization of glucohol sensors for on‐body operation. A) Schematic diagram representing the time course for operation of an iontophoretic‐sensing tattoo device, containing meal and alcohol consumptions, iontophoresis, and amperometric sensing of glucose and alcohol. B,D) Amperometric response of glucohol biosensor under experimental conditions depicted in (A) for human subject 1 and human subject 2 with consumption of meal and 355 mL of beer. Biosensor signal measured before and after consumption of alcohol and meal. C,E) Amperometric response of glucohol biosensor in control experiments without consumption of alcohol and meal for human subject 1 and subject 2. Amperometric response of the glucohol biosensor (under the experimental conditions depicted in (A) but without F) the enzyme immobilization or G) iontophoresis). For all experiments, the amperometric measurements were conducted in phosphate buffer solution, pH = 7.4, for the duration of 60 s and the step potential of −0.2 V (vs Ag/AgCl). Insets: Corresponding blood concentration of glucose and breath alcohol concentration.

Based on the selective and sensitive response of the glucohol tattoo toward the target glucose and alcohol biomarkers, we proceeded with on‐body testing using human subjects (**Figure** **4**). The epidermal demonstrations were conducted with recruited healthy volunteers under their informed written consent. These on‐body tests involved the consumption of food and alcoholic beverages to induce spikes in blood glucose and alcohol levels, respectively, expected in real‐life scenarios (Figure 4A). The results shown in Figure 4B,D were obtained using two healthy male subjects (subject 1 and 2) with the consumption of a meal and alcoholic beverage. Control evaluations carried out without such meal or alcohol uptakes (keeping fasting state), are shown in Figure 4C,E. Subjects 1 and 2 showed steep rise in their blood glucose and alcohol levels after the ingestion of food and drinks (as measured by commercial glucometer and breath analyzer). The epidermal glucohol sensor displayed a similar trend of increased ISF glucose and sweat alcohol signals. In contrast, no change in the signal of either sweat alcohol or ISF glucose was observed when food and alcohol were not ingested, reflecting a continued fasting state. Additional control experiments were carried out without the enzyme immobilization and without IP, as shown in Figure 4F,G, respectively. As expected, these control tests displayed consistent negligible current signals, despite the elevated blood glucose and alcohol levels by consumption of food and alcohol. These control experiments indicated that IP played a critical role in sampling the noninvasive biofluids (ISF, sweat) and that the presence of enzyme was required for biosensing the collected analyte. Clearly, the measured glucose and alcohol signals originated from the extracted ISF and stimulated sweat samples. The immobilized enzymes (GOx, AOx) ensured a selective response for detection of glucose and alcohol in the extracted ISF and generated sweat, respectively.

### 2.8 Human Subject Studies with Varied Meal/Alcohol Consumption Courses

**Figure 5:**
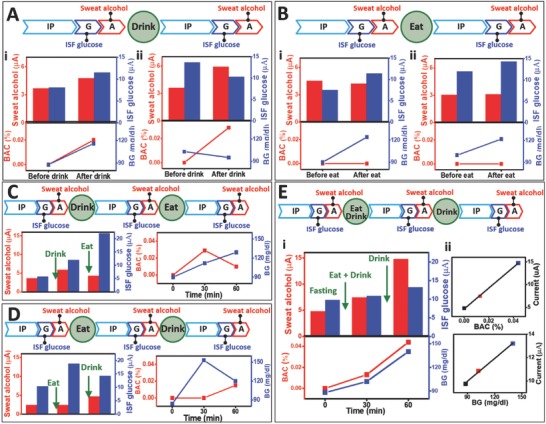
Glucohol biosensor sensing performance with varying meal/alcohol intake courses. Experimental time course and amperometric response of glucohol biosensor with measured concurrent concentrations of blood glucose and breath alcohol with A) alcohol intake and no meal for (i) human subject 1 and (ii) human subject 2, B) meal and no alcohol intake for (i) human subject 1 and (ii) human subject 2, C) alcohol intake followed by meal, D) meal followed by alcohol intake, and E) meal and first alcohol intake followed by second alcohol intake with experimental data and correlated epidermally measured glucose and alcohol concentrations with measured blood and breath concentrations (i). Corresponding blood glucose concentrations and breath alcohol concentrations (ii). For all experiments, the amperometric measurements were conducted in phosphate buffer solution, pH = 7.4, for the duration of 60 s using a step potential of −0.2 V (vs Ag/AgCl).

For better understanding the potential of the glucohol sensor in realistic scenarios, additional on‐body testing strategies were established by varying the order of the meal and drink consumption (**Figure** **5**). The first procedure in this variation involved only drinking an alcoholic beverage. As shown in Figure 5A, in case of subject 1, an alcoholic drink was consumed at fasting state leading to increased alcohol and glucose levels in sweat and ISF, respectively, as well as in blood. The increased glucose levels reflected the sugar content of the alcoholic drink. On the other hand, subject 2 showed a different glucose profile after alcohol consumption, with decreased ISF glucose and BG values, along with increased sweat and blood alcohol (Figure 5A‐ii). This result is attributed to physiological insulin action. Since the subject's BG value was 103 (higher than fasting BG level for healthy subjects), the already active insulin apparently acted to reduce the BG level to 95 mg dL^−1^. The ISF glucose signal displays a similar trend (i.e., a decrease after the alcohol consumption).

In the second alternate protocol, a meal was solely consumed without any alcoholic drinks. As expected, both subjects 1 and 2 maintained a blood alcohol concentration (BAC) of 0.00%, as measured by a commercial breath analyzer and the sweat alcohol response. The meal consumption induced glucose spikes (i.e., increase in BG) in both subjects, and the ISF glucose response was consistent with the observed BG value (Figure 5B). It should be noted that although subject 2 had a BG of 100 before meal consumption, the BG and ISF glucose increased after meal, indicating the dominant role of the glucose uptake despite the insulin effect (Figure 5B). These insulin effects may have different outcomes among individuals, also depending on the amount of sugar in the consumed food.

Next, a meal and alcohol were sequentially consumed in different orders. The first course of this sequence was designed to have alcohol intake followed by meal intake (Figure 5C). The BAC level spiked initially due to alcohol consumption, followed by decreased BAC due to metabolic alcohol degradation. The sweat alcohol response corresponded with the BAC response. In the case of glucose response, sequential alcohol and meal consumption increased the BG continuously, as well as the ISF glucose response (Figure 5C). The gain in BG value following the alcohol intake again reflects the sugar content of the drink. A subsequent consumption of a sugar‐rich meal led to a continuous increase in BG and ISF glucose. This behavior is consistent with the BG profile shown in Figure 5B. A different scenario involved a meal intake before the alcohol consumption. A glucose‐rich meal caused a steep spike in BG only, while retaining 0.00% BAC (Figure 5D). The signals of both ISF glucose and sweat alcohol followed the trend observed for blood. Subsequently, alcohol consumption (with less sugar than the meal) was carried out. The alcohol signal in both blood and sweat increased, while the glucose response in blood and ISF decreased. This is in agreement with Figure 5A subject 2 where a small amount of glucose intake (in the alcoholic beverage) is buffered by active insulin.

In order to better demonstrate the applicability of our glucohol sensor in real‐life scenarios, the final procedure evaluated involved the simultaneous consumption of a single meal with an alcoholic beverage followed by a subsequent second alcoholic beverage intake with no additional meal. Multiple measurements were taken throughout this procedure, tracking the temporal glucose and alcohol profiles (Figure 5E). Repeated alcohol consumption in a short period of time could lead to continuous increments of one's blood alcohol level, easily reaching dangerous vehicular situations (such as driving under the influence, DUI) and health issues (such as alcoholism). As demonstrated in Figure 5E, both ISF and blood glucose levels increased with the meal and the first alcohol consumption, and both glucose concentrations increased further following the second alcohol consumption. This trend differs from previous results showing decreased BG when alcohol was consumed in the presence of high BG, reflecting the subject's individual glucose metabolic system. The resulting BG level and glucose ISF biosensor signals display a linear correlation (*R* ^2^ = 0.998) using the same subject. The subject's alcohol levels in sweat and blood were also monitored sequentially upon the consumption of two alcoholic beverages, which led to a serial increase of BAC from 0.000, 0.013 to 0.044%, with proportionally increased sweat alcohol signals. The resulting plot correlating the blood‐alcohol level and sweat‐alcohol current signal, shown in Figure 5E subject 2, displays an excellent linear correlation (*R* ^2^ = 0.999). It should be noted that in all cases, ISF glucose and sweat alcohol readings from the wearable glucohol device showed similar trends with the blood levels measured by the commercial (glucometer and breath analyzer) readers. Such good agreement was confirmed by the corresponding correlation plots. These extensive on‐body data indicate the potential use of the epidermal sensors for simultaneous noninvasive glucose and alcohol measurements.

## 3 Conclusion

We have reported the first example of a dual epidermal fluids sampling and detection system, fully integrated onto a single conformal wearable platform. The design and operation of the new printed flexible device were optimized to ensure reliable and efficient iontophoretically stimulated simultaneous collection of the individual biofluids at the corresponding biosensor sites with no intersample mixing. Such rational design thus allows parallel sampling of the ISF and sweat biofluids toward noninvasive detection of different biomarkers. The dual fluid sampling and analysis concept was implemented using cost‐effective screen‐printing of body‐compliant, disposable temporary tattoo platform along with conformal wireless electronics. The new concept was illustrated for noninvasive glucose and alcohol analysis in healthy human subjects following meal and drink consumptions, with good correlations to commercial blood glucometer and breath‐analyzer devices. Future efforts will focus on large population studies for monitoring glucose and related alcohol effects in diabetes and prediabetes subjects. The new wearable sampling/sensing concept can be readily expanded to the monitoring of different biomarkers in the ISF and sweat fluids in connection to variety of healthcare and wellness applications. The simultaneous sampling and analysis of different noninvasive biofluids could thus pave the way for improved biomarker monitoring and enhanced assessment of a wearer's physiological status.

## 4 Experimental Section

*Chemicals and Instruments*: GOx (from *Aspergillus niger*, Type X‐S (EC 1.1.3.4)), AOx (from *Pichia pastoris*, 10–40 units mg^−1^ protein), chitosan, bovine serum albumin (BSA), potassium phosphate monobasic (K_2_PO_4_), potassium phosphate dibasic (K_2_HPO_4_), ethanol, pilocarpine nitrate, sodium nitrate, l(+)‐ascorbic acid, uric acid, agarose type IV, poly(vinyl alcohol) (PVA, average molecular weight = 70 000–100 000), and d(+)‐ glucose, were obtained from Sigma‐Aldrich (St. Louis, MO). Acetic acid was obtained from EMD Chemicals Inc. (Gibbstown, NJ). Polyurethane (PU) (Tecoflex solution grade, SG‐80A) was purchased from Lubrizol (Wickliffe, OH). All reagents were used without further purification. Electrochemical characterizations were performed at room temperature using a µAutolab type III PGSTAT302N (Metrohm), controlled by Autolab NOVA software v 1.11.2.

*Fabrication and Chemical Modification Process of Tattoo Biosensor*: The glucohol tattoo biosensor was fabricated using screen‐printing techniques (Figure 1B). The electrode array consisted of two IP electrodes (anode and cathode), two reference electrodes, and current collectors patterned from Ag/AgCl ink. Additionally, two working electrodes and two counter electrodes were patterned from PB ink. The entire electrode configuration was designed to resemble a panda with working and counter electrodes as eyes and ears, respectively. The patterns were designed in AutoCAD (Autodesk, San Rafael, CA) and outsourced for fabrication on stainless steel through‐hole 12 in. × 12 in. framed stencils (Metal Etch Services, San Marcos, CA). A sequence of the silver/silver chloride (Ag/AgCl) ink (4001, Engineered Conductive Materials, LLC, Delaware, OH), Prussian blue conductive carbon (C2070424P2, Gwent Group, Pontypool, UK), and insulator (Dupont 5036, Wilmington, DE) inks were patterned on Papilio temporary transfer tattoo base paper (HPS LLC, Rhome, TX) employing an MPM‐SPM semiautomatic screen printer (Speedline Technologies, Franklin, MA). First, Ag/AgCl ink was used to print the pair of iontophoretic electrodes, reference electrodes, and current collectors. Next, the working and counter electrodes (for glucose and alcohol detection) were printed using PB ink. After each step, the printed patterns were cured at 80 °C for 10 min in a convection oven. Finally, a transparent insulator was screen‐printed over the surface of the electrode pattern to confine the electrode and contact areas. Following the printing of the glucohol tattoo sensors, the working electrode located at the cathode compartment was functionalized with the glucose recognition layer. A volume of 1.5 µL of GOx 40 mg mL^−1^, containing 10 mg mL^−1^ BSA stabilizer, was mixed with 1.5 µL of chitosan solution (0.5 wt % in 0.1 m acetic acid) and the total volume (3 µL) was casted on the electrode. A similar protocol was used to functionalize the working electrode at the anode compartment with the alcohol recognition layer. A volume of 4 µL of AOx (as received) was mixed with 2 µL of a 10 mg mL^−1^ BSA stabilizer, and 2 µL of chitosan solution (0.5 wt% in 0.1 m acetic acid). Subsequently, 2 µL of PU was casted on top of the AOx layer. The total volume (8 µL) was casted on the working electrode surface. The enzyme modified glucohol tattoo biosensor was dried at 4 °C overnight.

For the dual IP process, agarose hydrogel was used at the cathode compartments, and PVA cryogel loaded with pilocarpine was used at the anode compartment. Agarose hydrogel was prepared by heating a continuously stirred agarose solution (2% w/v or 4% w/v) with 0.1 m potassium phosphate buffer (pH 7.0) until completely dissolved. The PVA cryogel was prepared as described in a previous report.^15^ Subsequently, the prepared cryogels (2.7 cm^2^) were soaked in 1% pilocarpine nitrate for 1 h. The enzyme modified glucohol sensor was prepared for dual IP as follows. First, 10 µL of the 2% dissolved agarose hydrogel was cast on the AOx‐modified working electrode. Next, 300 µL of agarose 4% was cast onto the working, reference, counter, and cathode IP electrode at the cathode compartment to cover 2.7 cm^2^. Finally, the PVA cryogel (2.7 cm^2^) loaded with 1% pilocarpine was placed on top of the reference, counter, anode IP, and AOx/agarose working electrode at the anode compartment shared the same PVA cryogel. Detailed depiction of the individual gel and electrode areas is presented in Figure S1 (Supporting Information).

*Tattoo Transfer Process and Mechanical Deformation Demonstrations*: The skin conformability and mechanical integrity of the glucohol biosensor were examined through repetitive bending, twisting, and stretching. To begin, a double‐sided adhesive was added to the screen‐printed electrodes (Figure 2A). The nonstick layer was then removed and the tattoo biosensor was applied to the upper arm of a human subject as a common temporary tattoo by gentle application and wetting. Subsequently, the skin‐worn tattoo was subjected to iterations of mechanical deformation and examined for apparent cracking or degradation (Figure 2B). A similar examination process was applied to the glucohol biosensor connected to the flexible wireless PCB. Specifically, the integrated sensor and PCB were subject to repeated bending iterations and examined for cracking or degradation (Figure 2C).

*Optimization of Iontophoretic Operating Conditions*: The operational parameters of the IP step for sweat generation and glucose extraction were determined by applying various IP current and durations. Sweat generation was quantified by measuring sweat weight using filter paper. After iontophoresis at various conditions, the skin surface was wiped with clean paper towel, and filter paper was placed and sealed with plastic wrap for 5 min. The weight difference of the filter paper before and after placement on the skin was measured and interpreted as the total sweat amount generated. For optimization of the cathode compartment, extracted glucose was measured at various operational IP settings. Before IP, the amperometric response of glucose was recorded without the presence of ISF. After IP, the glucose signal was again obtained, the difference in the current response should be proportional to the amount of extracted glucose through IP.

*Evaluation of Sensor Performance in Buffer Medium*: The experiments to evaluate the performance of the alcohol and glucose biosensors were made in vitro using a µAutolab type III PGSTAT302N (Metrohm), controlled by Autolab NOVA software v 1.11.2. For the glucose in vitro experiments, a 0.1 m PBS (pH 7.4) was used. All amperometric responses were recorded after 1 min incubation in the sample solution, using a potential step of −0.2 V (vs Ag/AgCl) over 60 s. Glucose response was tested in the physiological range of glucose, up to 160 × 10^−6^ m with 20 × 10^−6^ m increments. The glucose sensor specificity was examined using 60 × 10^−6^ m glucose solution in the presence of relevant electroactive constituents. Namely, 10 × 10^−6^ m each of ascorbic acid, uric acid, and acetaminophen were added to the glucose containing solution to determine potential influences on the amperometric response. The electrochemical performance of the glucohol biosensor toward alcohol detection was evaluated in a 0.1 m PBS (pH 7.4). The amperometric responses were measured, after 1 min immersion in the test solution, by stepping the potential to −0.2 V (vs Ag/AgCl) for 60 s. Calibration plots were obtained using 4 × 10^−3^ m ethanol increment additions, up to 40 × 10^−3^ m. The ethanol selectivity was examined by measuring the responses using 10 × 10^−3^ m ethanol in the presence of relevant electroactive species such as 0.2 × 10^−3^ m glucose, 10 × 10^−3^ m lactate, 84 × 10^−6^ m creatine, 10 × 10^−6^ m ascorbic acid, and 60 × 10^−6^ m uric acid. The influence of pilocarpine upon the activity of the PB transducer was addressed by drop‐casting 2% agarose gel containing PBS on the AOx‐modified working electrode.^15^

*On‐Body Evaluation Protocol with Human Subjects*: Epidermal evaluation of the glucohol biosensor on human subjects was conducted in strict compliance following a protocol approved by the Institutional Review Board (IRB) at the University of California, San Diego, CA. A total of 11 healthy volunteers were recruited, under their informed written consent, for on‐body evaluation of the developed sensor before and after consumption of food and alcoholic beverages. The glucohol tattoo sensors were transferred to the deltoid of the volunteers, then the flexible PCB board was connected to the tattoo using magnets as in previous reports.^9^, ^15^ Briefly, circular magnets were attached to the extended connections of the device (Figure S2, Supporting Information) and attached to the rod magnets on the PCB board. Immediately before starting the experiment, blood glucose and alcohol levels were measured using commercial glucose strips (Accu‐Chek Aviva Plus) and a commercial FDA‐approved breath analyzer (Alcovisor Mars Breathalyzer, Hong Kong) to validate the sensor performance. Next, the dual IP was performed. Specifically, a mild current density of 0.3 mA cm^−2^ (0.8 mA total current) was applied to the skin through cathode and anode IP electrodes for 5 min in order to induce sweat (at the anode) and simultaneously extract ISF (at the cathode) (Figure 1E). Afterward, the amperometric ISF glucose response at an applied potential of −0.2 V (vs Ag/AgCl) was recorded for 5 min (in connection to five potential steps of 1 min; such operation is essential for reaching the analytically useful steady‐state response. Accordingly, only the last stable amperogram is presented). Then, similarly, the amperometric response of alcohol in sweat was recorded at an applied potential of −0.2 V (vs Ag/AgCl) for 5 min (five amperograms with 60 s each). After these initial responses were recorded, the subjects consumed sugar‐rich food with an alcoholic beverage (12 oz. of beer or 5 oz. of table wine) and waited for 15 min to allow glucose and alcohol diffusion in blood. Next, blood glucose and alcohol were measured for a second time, and the IP and sensing cycle was repeated. Dual IP was performed, followed by glucose ISF and sweat alcohol measurements in the same fashion as previously described. With the completion of the experiment, the glucohol sensor device was removed. In order to validate sensor performance and selectivity, three control experiments were devised. First, following the otherwise identical experimental conditions described above, subjects 1 and 2 were instructed to not consume any food or alcohol. Second, also under identical experimental conditions the sensor was left unmodified with GOx and AOx. The final control experiment was achieved by replacing both of the 5 min IP steps with 5 min of wait time to minimize unknown experimental variables, yet highlight the role of IP in generating biofluids.

In addition to the initial measurements, further experiments were carried out to determine glucohol sensor response over time and with varying chronologies of food/alcohol consumption. The first set of experiments involved only drinking alcohol without glucose intake. Identical to the previous on‐body demonstrations, 5 min of IP was applied and ISF glucose and sweat alcohol measurements were followed. Two subjects were asked to consume an alcoholic beverage and after 10 min a final IP/sensing step was carried out to determine the ultimate amperometric responses to glucose and alcohol. In a second set, the alcoholic drink was replaced with a meal while other parameters remained the same. Additional experiments further investigated sensor response involving sequential drinking and eating in different orders of consumption. While keeping the same procedure above of 5 min IP, sensing glucose and alcohol, followed by consuming an alcoholic drink and a 10 min wait time, then IP, sensing cycle repeated. In another set of experiments, the order of consumption was switched first to a meal, followed by an alcoholic beverage. Furthermore, an additional experiment was carried out to determine the feasibility of detecting sequential alcohol consumption with a singular meal intake following the same procedure above of IP for 5 min, sensing glucose and alcohol, followed by a first alcohol/meal measured. A second alcoholic beverage with no food consumed, followed by an additional 10 min wait time and a final IP/sensing step was carried out to determine the ultimate amperometric responses to glucose and alcohol.

*Wireless Electronics*: A wireless electronic instrumentation circuit was developed to apply the iontophoretic current to the biosensor patch, noninvasively extract glucose and alcohol molecules, to measure the resulting concentrations via constant‐potential amperometry, and wirelessly deliver measured results to a smartphone or laptop for further processing and/or analysis. The PCB was designed on a 177 µm polyimide flexible substrate to conform to the shape of the body. The board included a Texas Instrument CC2541 2.4‐GHz BLE and Proprietary System‐on‐Chip device, a Texas Instruments LMP91000 Configurable AFE Potentiostat, and several DC‐DC converters for voltage supply regulations to generate reference potentials (Figure S3A, Supporting Information). Additionally, a 3‐Terminal Adjustable Current Source (LM334) was selected to provide the required iontophoretic current of 0.8 mA.

The iontophoretic current and constant‐potential amperometry measurements needed to be completely isolated to avoid unintended current loops in the electrode area. Therefore, an appropriate switching mechanism should be used to provide this isolation. To keep the board area small, a single potentiostat was used. Thus, a set of high‐voltage switches were needed to switch between the glucose and alcohol sensing electrodes during amperometry, as well as to enter a high‐impedance state during iontophoretic processing. Two Analog Device ADG452BRUZ chips were used to provide eight SPST CMOS switches for eight pins: three glucose amperometric pins, three alcohol amperometric pins, and two iontophoretic pins. In each mode (IP, glucose sensing, alcohol sensing), the BLE microcontroller set the switching modes accordingly (Figure S3B, Supporting Information). The power consumption of the board was provided using a CR2032 Lithium coin battery (Figure S2, Supporting Information). These power levels were measured to be 30 and 6 mW in iontophoretic and amperometric phases, respectively.

## Conflict of Interest

The authors declare no conflict of interest.

## Supporting information

Supplementary
