# Supplementary material for: Simultaneous Monitoring of Sweat and Interstitial Fluid Using a Single Wearable Biosensor Platform
Source: Adv Sci (Weinh). 2018 Aug 2;5(10):1800880. doi: 10.1002/advs.201800880 (PMC6193173; doi:10.1002/advs.201800880)
Supplement: Supplementary file 1 — Supplementary [file ADVS-5-1800880-s001.pdf]

Copyright WILEY-VCH Verlag GmbH & Co. KGaA, 69469 Weinheim, Germany, 2018.

ADVANCED SCIENCE
Open Access

Supporting Information

for *Adv. Sci.,* DOI: 10.1002/advs.201800880

Simultaneous Monitoring of Sweat and Interstitial Fluid Using a Single Wearable Biosensor Platform

Jayoung Kim, Juliane R. Sempionatto, Somayeh Imani, Martin C. Hartel, Abbas Barfidokht, Guangda Tang, Alan S. Campbell, Patrick P. Mercier, and Joseph Wang**

Copyright WILEY-VCH Verlag GmbH & Co. KGaA, 69469 Weinheim, Germany, 2016.

Supporting Information

Simultaneous Monitoring of Sweat and Interstitial Fluid using a Single Wearable Biosensor Platform

Jayoung Kim[a], Juliane R. Sempionatto[a], Somayeh Imani[b], Martin C. Hartel[a], Abbas Barfidokht[a], Guangda Tang[a], Alan S. Campbell[a], Patrick P. Mercier[b], Joseph Wang[a]**

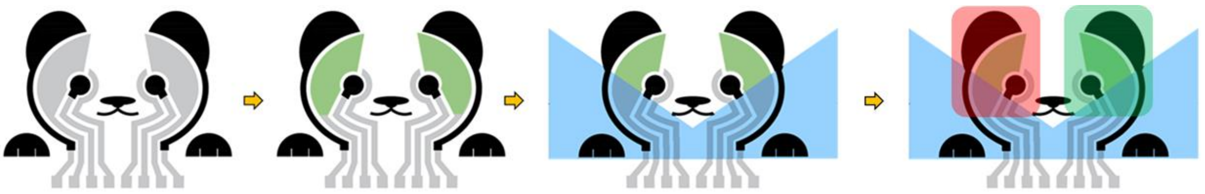

Figure S1. Schematic representation of *glucohol* sensor layout detailing the relative area of the IP electrodes at each step of fabrication. From left to right: *glucohol* patch as prepared by screen printing with IP electrodes highlighted in green having an area of 70 mm^2 each. Upon printing of the insulator layer (blue), the exposed area of the IP electrodes is reduced to 50 mm^2 each, which is the IP electrode surace area that delivers the applied current. The active area of the IP electrodes is then covred by a pilocarpine-loaded PVA gel (anode) or PBS-containing agarose gel (cathode), each having an area of 2.7 cm^2. The area of the skin where sweat is generated and ISF extracted is the same as the IP gels.

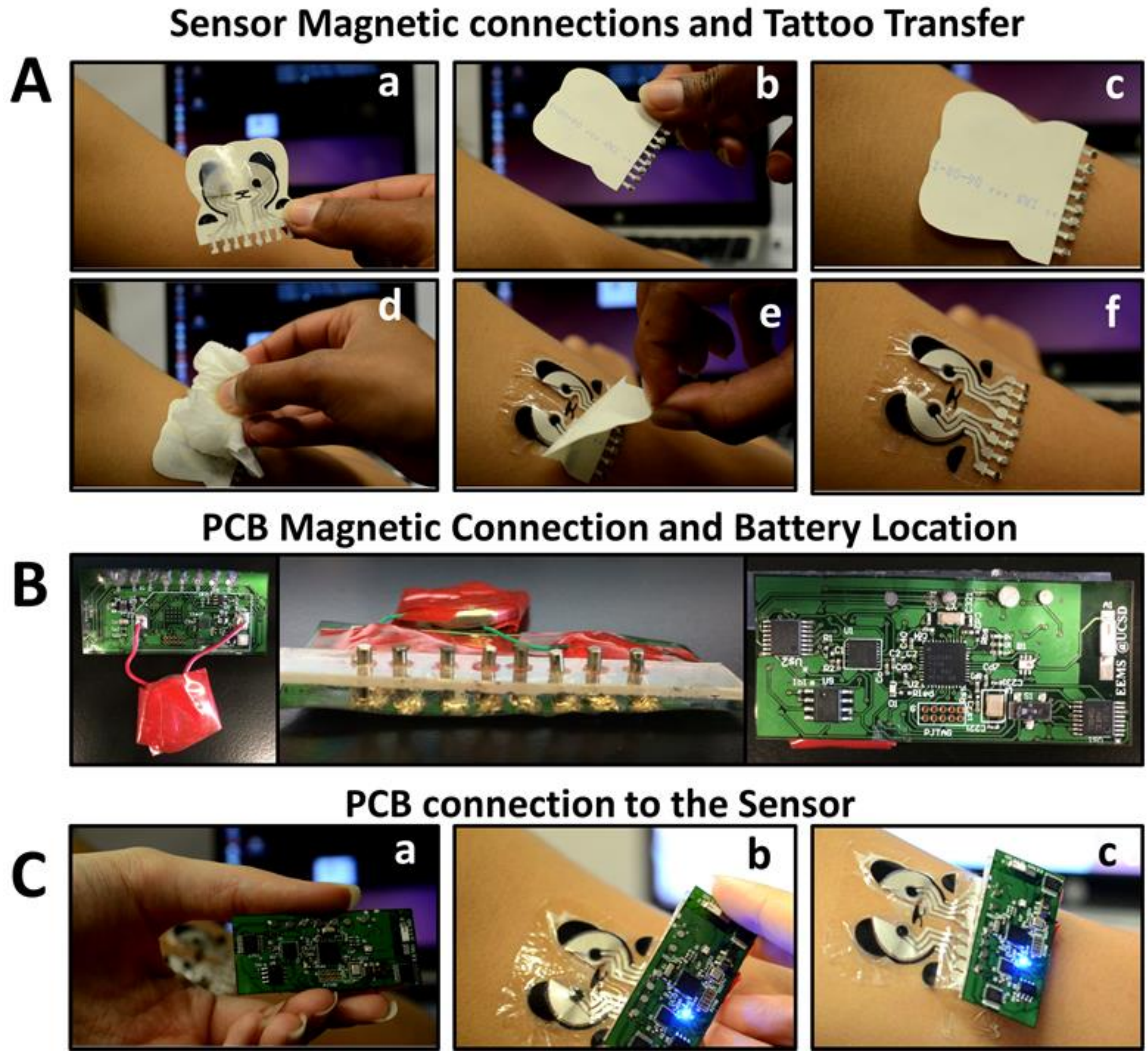

Figure S2: Tattoo transfer and PCB board integration for glucose and alcohol detection. A) Steps showing the process used to transfer the tattoo biosensor onto the skin. (a) Magnetic connections attached to the electrodes. Cathode and anode covered with PVA gel loaded with pilocarpine (left) and agarose gel (right), respectively. (b-c) Transferring the tattoo to the skin. (d-e) Releasing transferring paper using wet tissue. (f) *Glucohol* tatto sensor on the skin. B) Electronics used to perform IP and chronoamperometry for glucose and alcohol detection. From left to right: Coin battery attached to the PCB, Magnets attached to the PCB's pad contact using a flexible plastic spacer to avoid the magnets to collapse. Front view of the PCB with magnets and battery on the back side. C) Magnetic attachment of the PCB board to the transferred tattoo.

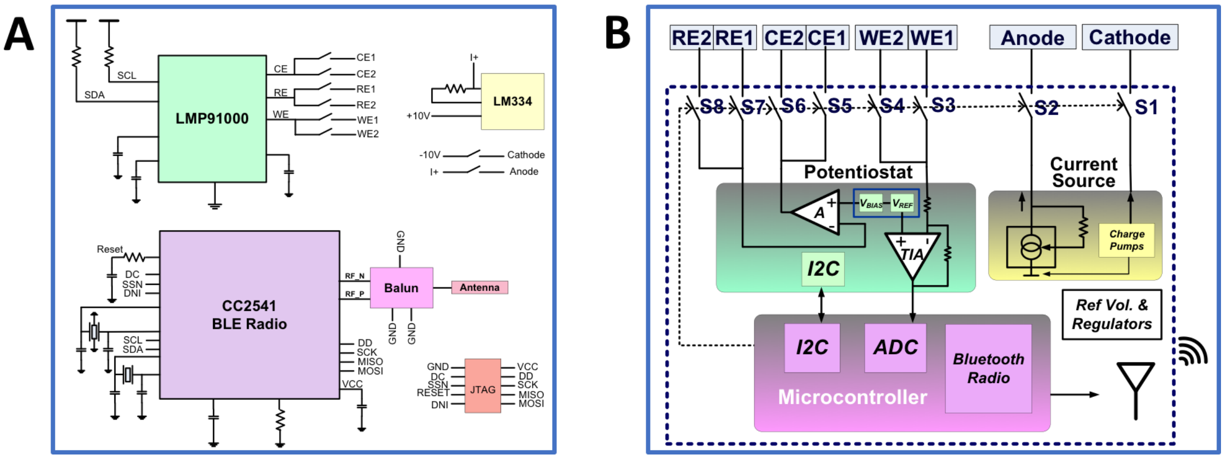

Figure S3. Wireless instrumentation electronic board. (A) Simplified schematic of the circuit. (B) A simple block diagram of the instrumentation electronic board.
